# Functionalized Gelatin/Polysaccharide Hydrogels for Encapsulation of Hepatocytes

**DOI:** 10.3390/gels10040231

**Published:** 2024-03-28

**Authors:** Christian Willems, Fangdi Qi, Marie-Luise Trutschel, Thomas Groth

**Affiliations:** 1 Department of Biomedical Materials, Institute of Pharmacy, Martin-Luther University Halle-Wittenberg, 06120 Halle, Germany; c-willems@gmx.de (C.W.); qfd4224168@gmail.com (F.Q.); 2 Department of Pharmaceutical Technology, Institute of Pharmacy, Martin-Luther University Halle-Wittenberg, 06120 Halle, Germany; 3 Interdisciplinary Center of Materials Science, Martin-Luther University Halle-Wittenberg, 06120 Halle, Germany

**Keywords:** alginic acid, hyaluronic acid, gelatin, hydrogels, hepatoblastoma cells, 3D culture, tissue models

## Abstract

Liver diseases represent a considerable burden to patients and healthcare systems. Hydrogels play an important role in the engineering of soft tissues and may be useful for embedding hepatocytes for different therapeutic interventions or the development of in vitro models to study the pathogenesis of liver diseases or testing of drugs. Here, we developed two types of hydrogels by crosslinking hydrazide-functionalized gelatin with either oxidized dialdehyde hyaluronan or alginate through the formation of hydrazone bonds. Gel formulations were studied through texture analysis and rheometry, showing mechanical properties comparable to those of liver tissue while also demonstrating long-term stability. The biocompatibility of hydrogels and their ability to host hepatocytes was studied in vitro in comparison to pure gelatin hydrogels crosslinked by transglutaminase using the hepatocellular line HepG2. It was found that HepG2 cells could be successfully embedded in the hydrogels, showing no signs of gel toxicity and proliferating in a 3D environment comparable to pure transglutaminase cross-linked gelatin hydrogels used as control. Altogether, hydrazide gelatin in combination with oxidized polysaccharides makes stable in situ gelling systems for the incorporation of hepatocytes, which may pave the way for use in liver tissue engineering and drug testing.

## 1. Introduction

In vitro models combining cells with extracellular matrix components in a 3D environment have been established to achieve a better understanding of the effect of matrix composition and stiffness on the growth and differentiation of cells but also for the establishment of cell co-cultures [1,2]. Such systems may be useful in engineering different soft and hard tissues for therapeutical applications. In addition, they can help to obtain a better understanding of tumor stroma cell interaction and to perform in vitro studies on the effect of drugs on human cells, better representing the in vivo situation and reducing the number of animal experiments [3].

Hydrogels are a potential way to fabricate 3D systems that represent different types of human tissue [3,4]. Hydrogels can be manufactured from synthetic or biopolymers, as both types have their advantages regarding stability, mechanical properties, and bioactivity [5]. Polysaccharides are the most abundant biopolymers in nature and because of their plentiful availability, relatively low production costs, and particularly their inherent bioactivity, they represent ideal candidates for making hydrogels and scaffolds to be used in tissue engineering [6]. Because of the presence of different functional groups, such as hydroxyl and carboxyl groups, polysaccharides can also be easily chemically modified to add specific functions to tailor their properties to different applications [7,8,9].

Hyaluronic acid (HA), for example, is a component of the extracellular matrix in mammalian tissues. HA has a wide range of functions, including activation of cell migration and growth, but also anti-inflammatory properties [1,10,11]. This is based on its interaction with specific cell-surface receptors, such as CD44, RHAMM, and others found in many cells [1]. Moreover, its swelling, dampening, and lubricating properties make it an essential component in tissues like the dermis and cartilage, as well as synovial fluid [12]. Particularly for the formation of hydrogels, its hydrophilicity, which leads to the uptake of large amounts of water related to its function in connective tissue, makes it highly interesting for biomedical applications [1,12]. Therefore, HA has been frequently used as a component of hydrogels [7] and in different pharmaceutical applications [13,14,15].

Alginic acid is another example of a polysaccharide frequently found in biomedical applications. It is prepared from brown algae and is a low-cost material. It is also biocompatible and biodegradable [16]. It can easily form hydrogels through the addition of Ca^2+^ ions and the subsequent formation of complex ion bonds between positively charged calcium ions and the negatively charged carboxyl groups of the guluronic blocks of alginate [17]. However, it lacks any bioactive cues to interact with cell-surface receptors and other proteins that regulate cell activities; hence, it represents a bioinert material. For applications that require bioactivity towards cells, alginate is therefore sometimes chemically modified to introduce some bioactive cues addressing cell surface receptors, particularly integrins [18] or combined with other extracellular matrix components like proteins or GAGs, to achieve a desired biological response and processability (for example, as bioink [19]).

Gelatin is a protein derived from bovine or porcine collagen that is biocompatible and bioactive. Gelatin is cheap and easily available and has been extensively investigated for a variety of biomedical applications [20]. It possesses essential cell-binding motifs like an arginyl-glycyl-aspartic amino acid sequence (RGD) interacting with integrin cell surface receptors, while metallo-matrix-proteinase-sensitive sites make the protein degradable [21]. There are still some drawbacks regarding the application of gelatin in tissue engineering, because, at 37 °C, it is a liquid and requires crosslinkers to form hydrogels, which can be also partly cytotoxic [22].

Liver diseases like cirrhosis, liver cancer, or inherited diseases still represent a major challenge in clinical care [23]. For some of these medical problems, liver transplantation still represents the best therapeutic option but is limited by the scarcity of donor organs [24]. Therefore, other therapeutic options have been discussed, ranging from wearable to implantable devices in the field of organ support [25]. Early work has discovered that the conventional 2D culture of human primary hepatocytes leads to the rapid decay of their functionality, while embedding them in a sandwich of collagen could maintain their function for up to several weeks. This is because the extracellular matrix that is in contact with hepatocytes is composed of collagen I, III, and IV, laminin, fibronectin, and different glycosaminoglycans like hyaluronan [26]. Hence, the use of matrix components as a substrate for the 2D and 3D culture (e.g., inside hydrogels) of hepatocytes has been applied with success [27,28]. In particular, embedding hepatocytes in hydrogels seems to be a promising option to maintain their functional activity over longer periods of time [28,29]. Nowadays, primary human hepatocytes or those derived from induced pluripotent stem cells may pave the way for different therapeutical interventions like engrafting them in the liver tissues of patients who lack specific enzymatic activities, but also for the development of in vitro models for studying diseases like cancer or for testing of drugs [30,31]. Besides human primary hepatocytes, different liver cell lines like the HepG2 hepatoblastoma cell line have been used for clinical applications like in bioartificial organs or for studies of biocompatibility of materials [32,33]. HepG2 hepatocytes possess many of the functions of primary cells, such as playing a role in the activity of different P450 cytochromes, urea, and albumin synthesis, which also makes them interesting for the development of in vitro systems studying the effect of material composition on hepatocyte growth and function [34].

Recent studies have investigated the use of gelatin/polysaccharide hybrid hydrogels in the field of tissue engineering and cell modeling. The combination of both materials leads to gels that can imitate the extracellular matrix (ECM) and provide bioactive cues to the cell for directed behavior [35]. Gelatin can form hydrogels with many different partners, such as cellulose, chitosan, alginate, and hyaluronic acid, for different applications. To form crosslinks between both components, different functionalization methods can be employed. For example, the modification of hyaluronic acid with diazirine groups leads to photo-cross-linkable, biocompatible, hybrid hydrogels with shear-thinning properties [36]. A widely used method is the Schiff base formation between the amine groups of the gelatin and aldehyde groups of functionalized polysaccharides. This has the benefit of abstaining from potentially toxic crosslinking agents. However, this formation is reversible and can be unstable. An alternative would be the usage of hydrazide groups instead, as the hydrazone bond formed with aldehyde groups is regarded as more stable [37]. Such hydrogels can form quickly and can find different applications. For example, hydrazone crosslinked hydrogels based on hyaluronic acid and gelatin were investigated for use as hemostatics [38].

**Figure 1:**
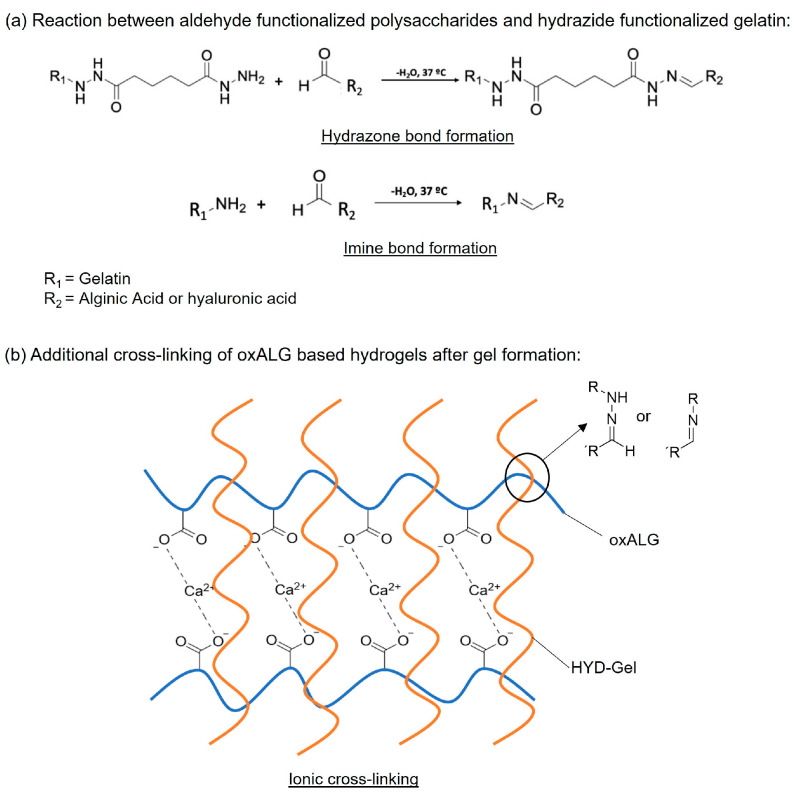
(**a**) Scheme of different chemical crosslinking mechanisms in the hydrogel formation. (**b**) Scheme of ionic crosslinking in hydrogel systems containing dialdehyde-functionalized alginate.

In this initial study, we investigated the formation of hydrogels based on dialdehyde-functionalized (bioactive) hyaluronic acid (oxHA) or (bioinert) alginic acid (oxALG) combined with hydrazide-functionalized gelatin (hGel). The hydrogel formation is primarily based on the reaction of hydrazide with aldehyde groups to form covalent hydrazone bonds, but also imine bond formation (with amine groups present in native gelatin) as a potential side reaction [36]. Besides the synthesis of the compounds, different ratios of oxidized polysaccharides with hGel were established and studied regarding their stability and mechanical properties to be applicable for embedding HepG2 cells. In the case of alginate-based systems, Ca^2+^ ions were added in a second step to provide this system with additional stability and strength [37]. A survey on the chemical and physical crosslinking reactions is shown in Figure 1. Initial studies on the cytotoxicity and biocompatibility of these hydrogels were performed with hepatoblastoma cell line HepG2, showing that some of the formulations provided excellent conditions for hepatocyte survival and growth. The results are reported herein.

## 2. Results and Discussion

### 2.1. Degree of Functionalization of Modified Gelatin and oxPS

Gelatin, hyaluronan, and alginate were chemically modified for crosslinking using reported methods [7,39]. A ^1^H-NMR of the hGel was recorded (Figure SI-1). Signals at 1.5 and 2.2 ppm appeared in the spectrum after the functionalization, which can be assigned to the CH_2_-groups of the adipic acid dihydrazide (ADH), thus confirming the success of the reaction. The functionalization degree of each component is shown in Table 1.

The oxidation degrees of polysaccharides (oxPS) were comparable with those of previous studies on the preparation of oxHA (12.5%/12.5%) and oxALG (20.7%/20.3%) [7,40]. The substitution degree of 48.6% for hydrazide groups in hGel in this study was slightly higher but generally in agreement with that of a previous study (43%) [39].

Different precursor concentrations of hGel and oxPS were investigated to find a suitable composition concentration for hydrogel formation. Initial studies conducted here have shown that the oxPS component must have a minimal concentration of 25 mg/mL and the hGel component a minimal concentration of 80 mg/mL, or there will be no gel formation. Degradation experiments with different mixtures (oxPS (mg/mL): hGel (mg/mL) = 25:80, 50:80, 25:160) were performed and demonstrated that these gels degraded very fast under physiological conditions (Figure SI-2). The oxHA-based gels were only stable for a maximum of 3 days, while an oxALG-based gel with the composition oxALG = 50 mg/mL and hGel = 80 mg/mL was still present after 10 days, but with a high loss of material (remaining wet mass < 50%). Therefore, the hGel component concentration was increased to 160 mg/mL and all following studies were performed with an oxPS concentration of 50 mg/mL and an hGel concentration of 160 mg/mL dissolved in PBS.

### 2.2. Mechanical Properties and Crosslinking Kinetics of Hydrogels

**Figure 2:**
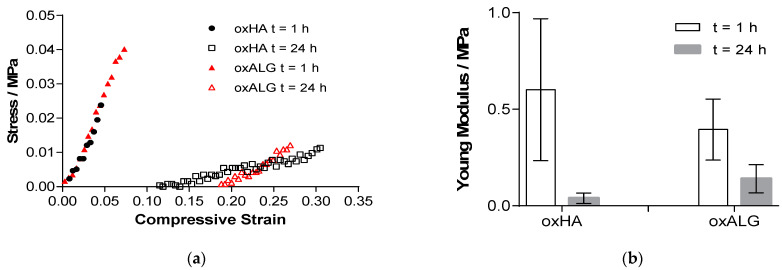
(**a**) Stress-strain diagram of the linear slope of selected hydrogel samples 1 h after synthesis and 24 h after incubation in a cell culture medium. (**b**) Young’s modulus of the hydrogels before and after incubation in cell culture medium (the hydrogels comprised hydrazide-functionalized gelatine (hGel) and either dialdehyde-functionalized hyaluronic acid (oxHA) or alginate (oxALG)).

The mechanical properties of hydrogels are important for cells, as they can affect specific biological reactions like growth and differentiation through mechanotransduction [41,42]. A texture analyzer was used to test the compression modulus of the hydrogels 1 h after their manufacture and 24 h after incubation in a cell culture medium (DMEM). The results are shown in Figure 2.

Both hydrogels displayed a compressive modulus that is comparable to native liver tissue (0.066 to 0.386 MPa), which makes them suitable for their use as substrates to culture or to embed liver cells [43]. Indeed, the oxALG gel showed a lower Youngs modulus after formation than oxHA, but also a smaller loss after incubation in a cell culture medium for 24 h, which suggested a better stability of these oxALG gels in a physiological environment.

**Figure 3:**
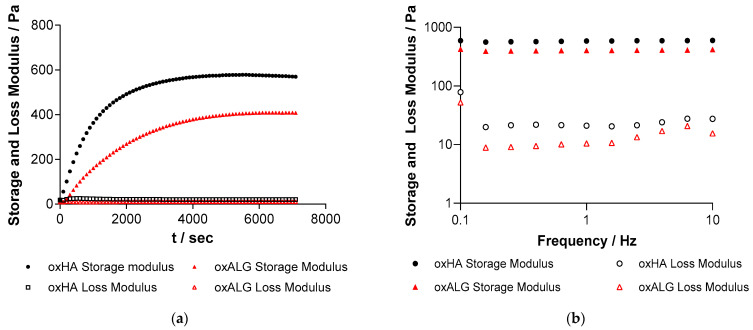
(**a**) Storage modulus and loss modulus of the hydrogel systems with either oxALG or oxHA (both c = 50 mg/mL) in dependence of time after mixing both precursors. T = 0 denotes the point of time when the oxPS and hGel (c = 160 mg/mL) are mixed. (**b**) Storage and loss moduli of the hydrogel systems depending on frequency. The measurement was taken immediately after the time-dependent measurement was finished. The data in the figure are representative of each system from 3 separate measurements.

Since the cell culture medium required for feeding cells encapsulated in hydrogels can interfere with the crosslinking process due to the presence of amino acids reacting with aldehyde groups of oxPS, the time needed for a hydrogel to form most of its crosslinks was also assessed by rheometry before the addition of medium. The storage and loss moduli were measured in dependence of the time after mixing both components and are shown in Figure 3a.

The point of gelation is usually determined by the intersection of the storage and loss moduli [44], which, in this case, happens a few seconds after mixing the oxPS with hGel. However, it was observed that the storage modulus continued to rise for both oxPS-based gels afterwards for some additional time, which suggests that the crosslinking reaction was continuing. Therefore, the slopes of the storage modulus at the beginning and the end of the experiment were calculated and their intersection was determined as the time when the hydrogel is mostly formed, as suggested by Poveda-Reyes et al. [44]. The gelation time is displayed in Table 2, along with the final storage and loss moduli of the hydrogels.

The frequency sweep was also measured shown in Figure 3b and shows that the storage and loss moduli are independent of the frequency in the measured range, which is typical for an elastic gel [44].

It is evident from the kinetics of gel formation that most of the crosslinking reaction was finished 1 h after gel components were mixed with each other. Therefore, in subsequent studies, the hydrogels were incubated for 1 h after mixing before adding the cell culture medium. Generally, oxALG-based hydrogels crosslinked slower than oxHA-based systems and their storage and loss moduli were significantly lower, which means that the gels are macroscopically softer. Previous studies have shown that as the degree of aldehyde functionalization increases, the molecular weight decreases [7]. As oxALG has a higher degree of oxidation and the molecular weight of native HA was higher than that of ALG before oxidation, oxALG should have a shorter chain length than oxHA, as demonstrated in one of our previous studies [7]. Since chain length also plays a role in hydrogel strength and stability because chains form entanglements with each other, higher gel strength of oxHA-based hydrogels can be expected [45]. Therefore, this factor probably contributed to a higher storage and loss moduli of oxHA-based gels, even though the degree of aldehyde functionalization was slightly lower compared to that of oxALG [7]. To enhance the stability and strength of oxALG-based hydrogels further, 0,1 M CaCl_2_ solution was added after 15 min of incubation. The Ca^2+^ ions can form additional crosslinks with carboxylic groups of alginate [46]. The storage and loss moduli of liver tissue can vary depending on the condition of the liver and the testing method, but it can range from 0.2 to 2.0 kPa for the storage modulus and 0.05 to 0.9 kPa for the loss modulus [47]. The values for the hydrogels without any cells were comparable to those of this tissue, which suggests that the gels should be suitable for the cultivation of liver cells.

### 2.3. Stability of Hydrogels

**Figure 4:**
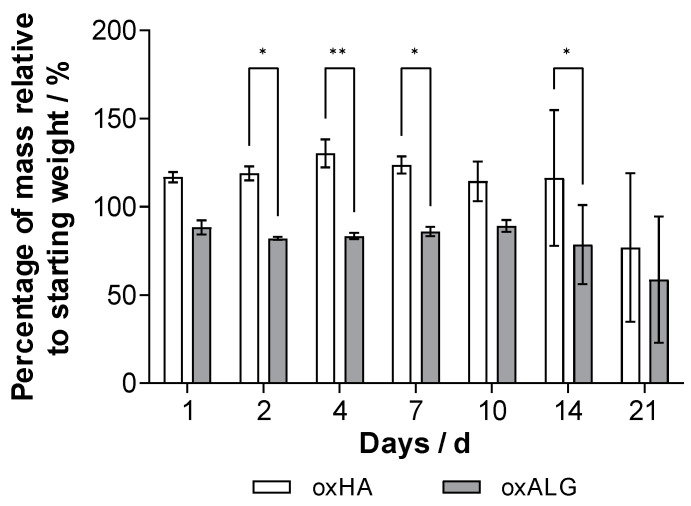
Change in hydrogel wet mass depending on time incubated in cell culture medium DMEM. Values represent the mean value and standard deviation. *n* = 4. * indicates statistical significance of *p* ≤ 0.05; ** indicates statistical significance of *p* ≤ 0.01.

**Figure 5:**
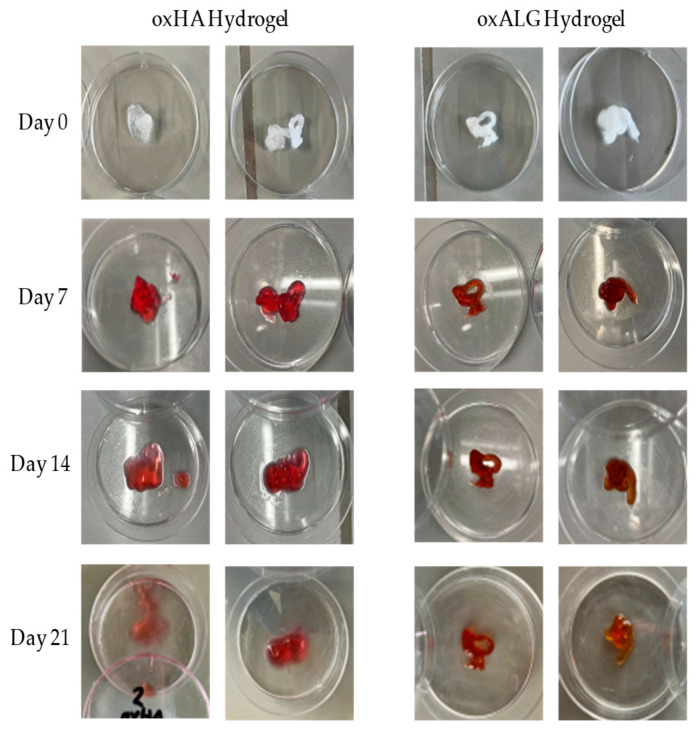
Photographs of two specimens of each type of hydrogel before (day 0) and after incubation in DMEM for up to 21 days. Left lanes: oxHA-based hydrogels, right lanes: oxALG-based hydrogels. Composition of hydrogels: oxPS: hGel = 1:1; c(oxPS) = 50 mg. c(hGel) = 160 mg/mL. The red color of gels is due to incubation in DMEM with phenol red.

To use hydrogels for tissue engineering or the embedding of liver cells and testing of drugs in vitro, it is important to determine their stability. Depending on the application, the hydrogels need to be stable for different amounts of time, but a stability of at least 7–10 days is needed for cell growth (e.g., the formation of spheroids) and corresponding matrix synthesis [48]. The stability of the hydrogels was tested by immersing them in a cell culture medium. The results of the study are shown in Figure 4, while photos of the gel shapes and their fidelity are shown in Figure 5.

The results in Figure 4 show that both types of gel are generally stable when immersed in cell culture medium DMEM for a period of up to 3 weeks. However, the oxHA gels swell strongly after 24 h due to the presence of hydrophilic groups, indicating that the forces of solvation exceed the intermolecular binding forces and suggesting that the crosslink density is not as high, so the hydrogel takes up additional liquid due to colloid osmotic pressure [49,50]. In contrast, the oxALG-based gels immediately lost about 12% of their initial mass but then were stable for up to 14 days. Although the amount of gel mass was higher in oxHA-based gels, the standard deviation was quite high and the shape fidelity of oxHA-based gels was much lower than that of the oxALG-based ones (Figure 5, left lanes. As can be seen in Figure 5, oxHA-based gels lost their form after 14 days. By contrast, in the Figure 5 right lanes, one can see that oxALG-based hydrogels keep their shape for up to 3 weeks of incubation in the medium. Due to the addition of CaCl_2_ solution, the gels made with oxALG possess additional crosslinks, which probably prevent the gel from taking up too much liquid after being immersed in the medium. A higher crosslinking degree generally leads to less swelling [51]. The loss of material is probably due to some uncrosslinked polymer chains being washed out during the first 24 h. Similar results were obtained by Boccacini et al., who observed swelling of the hydrogel followed by the washing out of uncrosslinked material depending on the degree of crosslinking [46]. Indeed, both types of hydrogel were stable for at least 14 days, which made it possible to use them in cell culture studies to analyze their ability to keep hepatocytes alive and growing.

### 2.4. Cell Culture Studies

To test the cytotoxicity of hydrogels, HepG2 cells were cultivated on the bottom of 96-well plates and covered with either hydrogel precursors or complete hydrogels. At first, they were cultivated in the presence of the gel precursors dissolved in PBS for 24 h. For the experiments involving only the single precursor solutions, the concentration that was chosen was identical to the one used for the preparation of hydrogels. The results shown in Figure SI-3a demonstrate that the solutions of hGel, oxHA, and oxALG were all toxic to cells because no viable HepG2 cells positively stained with the vital stain calcein could be seen in the confocal images, except the control (cells cultured in EMEM). All cells cultured in the presence of gel precursors were stained by nuclear stain ethidium bromide, indicating cell death. The absence of viability was also confirmed when the metabolic activity of cells was assessed with QBlue assay (Figure SI-3b) and no fluorescence signal was detected for HepG2 cells cultured in the presence of gel precursors. The reason for the toxicity of the hGel solution is probably related to the presence of hydrazide, as the unmodified gelatin is usually biocompatible. Hydrazide groups can react with proteins in aqueous media, which is a possible reason for the toxicity of the functionalized gelatin [52]. Regarding the toxicity of oxPS solutions, one can assume that polymer chain length has been severely reduced by oxidation through sodium periodate, which makes it easier for the compounds to be taken up by cells [7]. Since aldehydes can form adducts with nucleophilic sites on biological compounds inside cells such as enzymes and DNA, it will impair their function and cause cell damage like necrosis and apoptosis [53,54].

**Figure 6:**
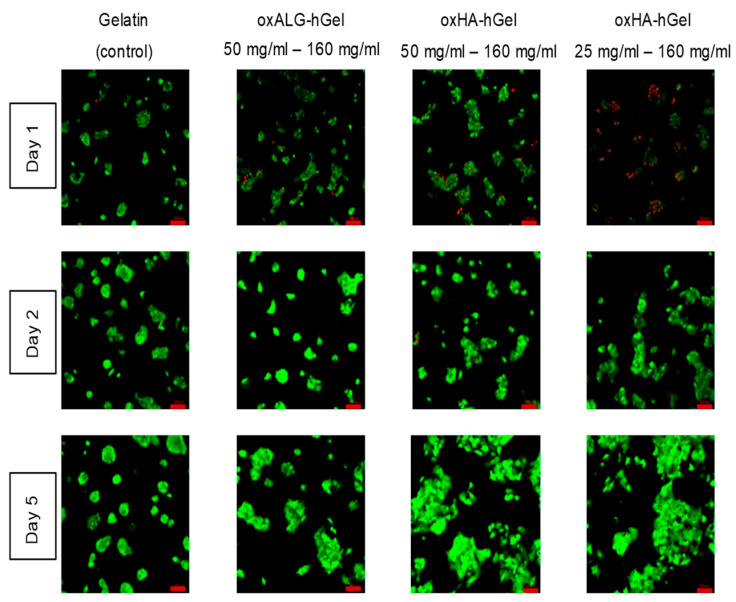
Confocal microscopy pictures of a live/dead staining assay of HepG2 cells, covered with either native gelatin crosslinked with transglutaminase used as a control or hydrogels composed of hGel and oxPS; Pictures display different times after the start of the experiment. Green channel: calcein, living cells; red channel: ethidium bromide, dead cells. Scale bar (red) = 100 µm.

The potential toxicity of the hydrogels after their formation was analyzed by seeding and preculturing cells on the bottom of the wells and then mixing the gel precursors hGel and oxPS so that hydrogels are formed on top of the cells. Here, gelatin crosslinked by transglutaminase according to an established protocol was used as a control for comparison [55]. Figure 6 shows the cultivated cells after different times of incubation underneath the hydrogels. Here, one example of the effect of a higher hGel-to-oxHA ratio with an oxHA concentration of 25 mg is also shown.

The images in Figure 6 analyze cell viability after 1, 2, and 5 days. Stronger toxicity can be seen after 1 day, when only 25 mg oxHA was used for gel formation, which indicates that some hydrazide groups of hGel did not take part in the crosslinking reaction. This negative effect was not observed when 50 mg oxHA or oxALG was used for gel formation. In general, both types of oxPS-based hydrogels allow for not only the survival of HepG2 cells but also their growth on the surface of tissue culture plates during subsequent days, which is evident by the increasing size of cell clusters typical for proliferating HepG2 cells. This was also comparable to or even more pronounced in comparison with cells cultured underneath a gelatin hydrogel crosslinked by transglutaminase, which was applied here as a control hydrogel. The HepG2 cells grew in large clusters during the duration of the experiment, suggesting that oxPS-based gels are nontoxic. The toxicity that the individual components showed was not present if the cells were covered by the gels. This is certainly because the components crosslink with each other so that they are bound and cannot be taken up by the cells. A comparable explanation is also proposed by Boccacini et al. for similar gel systems [56].

**Figure 7:**
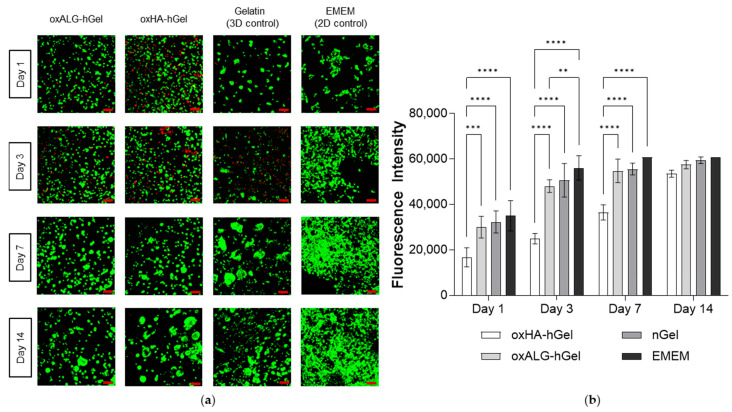
(**a**) Confocal microscopy pictures of a live/dead staining assay of HepG2 cells encapsulated in hydrogels made of either oxHA or oxALG mixed with hGel or native gelatin crosslinked by transglutaminase (nGel) used as a control. Photos display images of cells growing inside the gels visualized across the whole *z*-axis, compressed into one layer. Green channel: calcein (live cell stain); red channel: ethidium bromide (dead cell stain). Scale bar = 100 µm. (**b**) Results of a QBlue cell-viability assay of the HepG2 cells encapsulated in the hydrogels after different times of incubation. Three wells were prepared for each condition, and from each well, 3 samples were taken. Values represent the mean value ± standard deviation. ** indicates statistical significance of *p* ≤ 0.01; *** indicates statistical significance of *p* ≤ 0.001; **** indicates statistical significance of *p* ≤ 0.0001.

HepG2 cells were then encapsulated in hydrogels by adding the cell suspension to the hGel solution and immediately mixing it with the oxPS component in tissue culture plates. The viability and growth of HepG2 cells were evaluated at different points of time up until 14 days through live/dead staining and QBlue assay, and are shown in Figure 7a,b.

The CLSM micrographs displaying the results of cell staining experiments are compressed over the *z*-axis, showing the cells of different layers together in one picture. It was found that the cells were distributed evenly throughout the gel. The cell viability was compared with that of a 2D model of HepG2 cells grown on the bottom of a well plate in culture medium EMEM with 10% FBS and a 3D model of cells in transglutaminase- crosslinked gelatin. The latter was used as a control due to similarities in component makeup and known biocompatibility with hASC [53]. CLSM images show that, after 1 day, oxHA-based hydrogels still have a certain cytotoxicity indicated by red staining not seen in the other two gel formulations. In general, some dead cells were visible in all gel samples after 3 days of culture that were not observed in the 2D control. However, this was not seen after 7 or 14 days of culture. In particular, after 14 days in oxPS-based hydrogels, HepG2 cells grew in larger clusters compared to those in nGel control gels, which might be related to the more hydrophilic character of polysaccharide-based hydrogels. Studies with QBlue assay measuring the metabolic activity of cells after 1 day show clearly that the oxHA-based gels were related to lower cell viability than the 2D and 3D controls, while HepG2 cells cultured in oxALG-based gels showed a comparable fluorescence intensity to these controls. This was also maintained for the duration of the culture, while oxALG-based hydrogels supported the growth of HepG2 cells to a similar extent as the nGel-based hydrogel. As the mechanical properties of oxPS-based hydrogels are similar, the difference in cell viability and growth could be due to the different success of crosslinking with remaining nonbound aldehyde and hydrazide groups or differences in the molecular weight of the hyaluronic acid component. In our previous study, we also observed a higher toxicity of oxHA when compared to oxALG [7]. Accordingly, the higher cell viability and growth, especially of the oxALG-based systems, represent an improvement compared to previous studies of hydrogels based on oxidized cellulose sulfate in combination with carboxymethylchitosan; in those studies, the cells were also viable but did not have an incentive to grow [9]. The bioactive cues of the gelatin obviously stimulate cell growth, while the hydrazone bonds improve the stability of the gel in comparison with those based on only imine bond formation. The storage modulus of the hydrogel is also closer to that of hepatic tissue and stiffer than that of imine crosslinked gels, which stimulates cell growth further. This is in accordance with previous studies that observed lower cell growth in softer hydrogels compared to stiffer hydrogels [55].

The experiments show that the HepG2 cells are able to grow in hydrogels consisting of oxidized polysaccharides and hydrazide-functionalized gelatin. The oxPS-based systems, particularly oxALG, can support cell growth just as well as control systems made up of pure gelatin crosslinked by transglutaminase.

## 3. Conclusions

In this study, we investigated the potential of hydrogels composed of hydrazide-functionalized gelatin and aldehyde-functionalized polysaccharides for 3D liver cell culture. Our results demonstrate the feasibility of fabricating stable hydrogels with suitable mechanical properties for cell encapsulation. We found that higher polymer concentrations increase the stability of hydrogels while showing excellent biocompatibility towards hepatocytes. In vitro tests have proven that the gels can support cell growth and maintain viability over the span of several weeks, which makes them promising candidates for long-term in vitro studies. Since the gels form in the span of a few seconds and show mechanical properties beneficial to hepatic cell growth, an application in 3D bioprinting and tissue engineering is also possible. As the mechanical properties can be potentially further tuned, it is possible that the gels can be used for the cultivation of other cell types, such as stem cells. This also may open new possibilities in the manufacture of 3D tissue culture scaffolds for tissue engineering applications and in vitro test systems.

## 4. Materials and Methods

### 4.1. Materials

Gelatin type A (300 Bloom, Mw ≈ 50–100 kDa), adipic acid dihydrazide (ADH), 1-hydroxybenzotriazole (HOBt), sodium periodate (NaIO_4_), phosphate-buffered saline (PBS), and 2,4,6-trinitrobenzenesulfonic acid (TNBS) were purchased from Sigma-Aldrich (Taufkirchen, Germany). Unless noted otherwise, the PBS was used at a pH of 7.4. The *N*-(3-dimethylaminopropyl)-*N*′-ethylcarbodiimide hydrochloride (EDC), dimethylsulfoxide (DMSO), sodium hydroxide (NaOH), hydrochloric acid (HCl), calcium chloride (CaCl_2_), glycine, sodium dodecyl sulfate (SDS), and ethylene glycol were from Carl Roth GmbH (Karlsruhe, Germany). The sodium hyaluronate (Mw ≈ 1.2 MDa) was purchased from Kraeber & Co GmbH (Ellerbeck, Germany) and sodium alginate (Mw ≈ 236 kDa) was purchased from Thermo Fisher (Kandel) GmbH (Karlsruhe, Germany). The ethanol (99.8%) was purchased from AppliChem Panreac ITW Companies (Darmstadt, Germany). The hydroxylammonium chloride (NH_2_OH·HCl) and sodium hydrogen carbonate (NaHCO_3_) were purchased from Merck KGaA (Darmstadt, Germany). The dialysis membrane (Mw CO: 3.5 kD) was purchased from Thermo Fisher Scientific (Dreieich, Germany).

The hepatocellular carcinoma cell line 2 (HB-8065TM) (Hep G2) was purchased from ATCC^®^ (Manassas, VA, USA). The Dulbecco’s Modified Eagle’s Medium (DMEM) with a low glucose content of 1 g/L was purchased from HiMedia (Einhausen, Germany). The Eagle’s Minimum Essential Medium (EMEM) and glutamine were purchased from Carl Roth GmbH (Karlsruhe, Germany). The fetal bovine serum (FBS) and transglutaminase were purchased from Sigma-Aldrich. The penicillin/streptomycin/amphotericin B and glucose stock solution were purchased from Lonza (Köln, Germany). The trypsin was purchased from Biochrom (Berlin, Germany). The QBlue^®^ viability test kit was purchased from Biozol Diagnostic (Eching, Germany). The viability/cytotoxicity assay kit for live and dead animal cell detection was purchased from BIOTIUM (Fremont, CA, USA).

### 4.2. Synthesis of Hydrazide-Functionalized Gelatin

The synthesis of hydrazide gelatin (hGel) was performed using a previously reported method [39]. After the complete dissolution of 2 g gelatin in 200 mL of distilled water at 40 °C, the gelatin solution was cooled down to room temperature, 8.36 g of ADH was added to the solution, and the pH was adjusted to 5.0 with HCl. Next, 1.08 g of HOBt and 1.54 g of EDC were dissolved in 20 mL of a 1:1 mixture of DMSO and H_2_O. The mixture was added dropwise to the gelatin solution, while the pH was kept at 5.0. After the addition of the mixture, the reaction solution was stirred at room temperature for 3 h while keeping the pH at 5.0. The solution was stirred overnight at room temperature and was then dialyzed against distilled water for 3 days in a dialysis membrane with a cutoff of 3.5 kDa. The final product was analyzed by ^1^H-NMR spectroscopy (400 MHz at a Varian spectrometer) in D_2_O as a solvent. To determine the amount of hydrazide groups, a TNBS assay according to the literature was performed using adipic acid dihydrazide as the standard for the calibration curve [39].

### 4.3. Synthesis of Oxidized Hyaluronate (oxHA) and Oxidized Alginate (oxALG)

The oxidation of both polysaccharides (oxPS) was performed as follows. The synthesis of oxidized sodium hyaluronate was performed using a previously reported method [7]. Briefly, 1 g of sodium hyaluronate was dissolved in 200 mL Milli-Q H_2_O, and 0.68 g (1.2 molar equivalents in relation to 1 mol sodium hyaluronate) of sodium periodate was added to the solution and stirred overnight in the dark. Finally, 0.68 mL of ethylene glycol was added to the solution to quench the reaction. After dialysis with a membrane having a cutoff of 3.5 kDa for 3 days, the water was removed through lyophilization using a freeze dryer (Martin Christ, Osterode am Harz, Germany), and the product was obtained as a white powder. The synthesis of oxidized sodium alginate was performed using a previously reported method [57]. A total of 1 g of sodium alginate was dispersed in 10 mL ethanol, and 0.64 g of sodium periodate was dissolved in 10 mL of Milli-Q H_2_O and slowly added to the solution. The mixture was then stirred at room temperature for 6 h in the dark and quenched by adding 1 mL of ethylene glycol. After dialysis with a membrane having a cutoff of 3.5 kDa for 3 days, the water was removed through lyophilization and the product was obtained as a white powder.

The amount of aldehyde groups of oxPS was determined by titration. A mixture of 25 mL Milli-Q water (0.055 μS cm^−1^) and 20 mL of 0.4 mol L^−1^ hydroxylammonium chloride representing a blank was prepared and its pH was measured. This mixture served as the blank. Next, 60 mg of oxPS was dissolved in 25 mL of Milli-Q water and the pH of the solution was adjusted to 7.0 using NaOH (0.01 mol L^−1^). Next, 20 mL of 0.4 mol L^−1^ hydroxylammonium chloride was added and the reaction mixture was stirred for a minimum of three hours at room temperature. Sodium hydroxide (0.01 mol L^−1^) was used to titrate the released HCl until the pH of the blank was reached. Three replicates of each sample were titrated. The percentage of aldehyde content substitution was defined as oxidation degree. It stands for the molar amount of oxidated dimeric units divided by all dimeric units in biopolymers, as shown in (1).

Oxidation degree (%) = ((V × 0.01 × Mw)/2W) × 100(1)

Here, V is the volume of NaOH (V; unit [mL]), Mw is the molar mass of the HA dimeric units (379.32 g/mol) or ALG units (366.23 g/mol), and W is the weight of the sample (W; unit [g]).

### 4.4. Hydrogel Formation and Characterization

#### 4.4.1. Studies on Gel Stability

For all experiments, unless stated otherwise, the following mixing ratio was used: oxPS was dissolved at a concentration of 50 mg/mL in PBS, and hGel was dissolved at a concentration of 160 mg/mL in PBS at 37 °C. The mixing ratio between oxPS and hGel was 1:1. Both solutions were heated up to 37 °C and 0.2 mL of each solution was filled in a separate syringe. Both syringes were connected by short tubing and the mixtures were vigorously mixed for a few seconds and quickly dispensed in a previously weighed Petri dish. After incubating the gels for 1 h at 37 °C, the Petri dishes were weighed again and filled with DMEM culture medium and stored at 37 °C for different periods of time. For oxALG hydrogels, after 15 min of gelation, an additional crosslinking with CaCl_2_ was performed by submerging them in a prewarmed CaCl_2_ solution (0.1 M) for 45 min according to a protocol published previously, before it was replaced by DMEM [40].

At specific points in time, the DMEM was carefully removed, and the gel was weighed again. The weight change in percent was calculated as the difference between the starting weight (w_0_) and the weight at the time t (w_t_). Each sample had four replicates.

Change of hydrogel mass (%) = (w_t_ − w_0_)/w_0_ × 100 (2)

#### 4.4.2. Mechanical Properties of Hydrogels

The mechanical properties of hydrogels were characterized by a Brookfield CT3 texture analyzer (Brookfield, WI, USA). A total of 100 μL of 50 mg/mL oxPS solutions was carefully mixed with 100 μL of 160 mg/mL hGel solution in 1.5 mL tubes. The hydrogels were incubated according to the methods mentioned above. A 4 mm-diameter cylindrical stamp was compressed into the gel and released. The compression moduli of the fresh hydrogels and after 24 h immersion in DMEM were measured by compression mode at room temperature, with a 0.01 N trigger point, 0.2 mm/s test speed, and 0.2 mm/s back speed. The stamp depth H and corresponding force Fc encountered by the stamp with a flat blunt tip were noted. All samples were prepared in duplicate. The elastic modulus was determined by the slope of the stress-strain curve.

#### 4.4.3. Rheological Studies

The gelation of the hydrogels was monitored with a Kinexus Lab+ Rheometer (formerly Malvern, now Netzsch, Germany) with 20 mm plate-plate geometry for two hours. The lower plate was prewarmed to 37 °C. The hGel and oxPS solutions were prepared and warmed as mentioned before. Additionally, hGel with 0.1 M CaCl_2_ was prepared. First, 175 µL of hGel was placed in the middle of the lower plate. Then, 175 mL of oxPS was dropped centrally on the hGel. A gap of 1 mm was chosen and oscillation at 1 Hz and 1% started. Afterwards, a frequency sweep from 0.01 to 10 Hz at 1% shear strain were performed. Each sample had 3–5 replicates.

### 4.5. Biological Studies

The biological studies were performed with hydrogels consisting of an oxPS component with a concentration of 50 mg/mL, an hGel component with a concentration of 160 mg/mL, and a mixing ratio of both components of 1:1, as in all previous experiments. For biological studies, hepatocellular carcinoma cell line 2 (HepG2) was used (ATCC, Washington DC, USA). The cryopreserved cells were thawed and cultivated at 37 °C and 5% CO_2_ using EMEM containing 2 mM glutamine, 10% FBS, and 1% penicillin/streptomycin/amphotericin B. Cells were grown until they were nearly confluent and then detached with trypsin/EDTA for 5 min at 37 °C. Trypsin was stopped by the addition of FBS and then EMEM was added. Cells were centrifuged at 200 g and resuspended in EMEM with 10% FBS.

#### 4.5.1. Cell-Viability and Proliferation Studies

To evaluate the viability of HepG2 cells, a cytotoxicity assay kit was used. A total of 2.5 µL of 4 mM calcein AM solution and 20 µL of 2 mM EthD-III solution were added to 10 mL PBS and thoroughly mixed. The medium in the wells was removed and the wells were washed twice with PBS. The staining solution was added to the wells, and they were incubated for 1 h. Afterwards, the cells were analyzed using a CLSM 710 confocal laser scanning microscope equipped with ZEN 2012 software (Carl Zeiss, Oberkochen, Germany).

Cell viability and proliferation studies were also performed with samples of cells either covered by or encapsulated in the hydrogel (see below). The medium was removed and the wells were carefully washed with PBS 2 times. Afterwards, fresh medium containing 10% of QBlue reagent was added to each well and incubated for 4 h. Then, 100 µL of each solution was transferred into a black 96-well plate. The fluorescence of the samples was measured with a Fluostar Plate reader from OPTIMA, Offenburg, Germany (excitation wavelength: 544 nm, emission wavelength: 590 nm). The number of samples was *n* = 6.

#### 4.5.2. Cytotoxicity Studies by Coverage of HepG2 Cells with Hydrogel Components and Hydrogels

HepG2 cells were seeded in 48 well plates at a concentration of 30,000 cells/well for the cell viability assay and 20,000 cells/well for live/dead staining. Single hydrogel precursor solutions, oxPS, and hGel were dissolved in medium 24 h after the cell seeding, and 500 mL of these solutions was added on top of the cells for incubation. Here, 500 mL of cell culture medium was used as a negative control, and 20 mL of DMSO in 480 mL of medium as a positive control. The cytotoxicity of the hydrogels was studied by mixing 50 mL of oxPS and hGel. Then, the solutions were carefully added to each well on top of the cell layer. After 1 h of incubation at 37 °C to allow crosslinking, 500 mL of cell culture medium was added on top of the gels to allow for the nutrition of cells. The medium was changed after 24 h and then every second day. As a control hydrogel, 100 mL of unmodified gelatin (concentration 100 mg/mL) was mixed with 7.5 mL of transglutaminase (12 IU/mg; Sigma, Deisenhofen, Germany) and added to the wells. As a further untouched control, wells having only cells without gel coverage were used.

The viability and metabolic activity of cells were measured through live/dead cell staining and QBlue assay, respectively.

#### 4.5.3. Encapsulation of HepG2 Cells in Hydrogels

The oxPS was dissolved at a concentration of 50 mg/mL in PBS and the hGel was dissolved at a concentration of 320 mg/mL in PBS. Cells were suspended in a glucose solution containing 4.5 g/L glucose at a concentration of 4 × 10^6^ cells/mL. The cell suspension and the hGel solution were carefully mixed (1:1 ratio) so that the solution had a final hGel concentration of 160 mg/mL. The hGel with cells and the oxPS solution (50 µL of each) were taken up in a separate Eppendorf pipette. The solutions were carefully added to the well at the same time to ensure proper mixing of the components. After 1 h of incubation at 37 °C to achieve crosslinking, 500 µL of EMEM was carefully added to the wells to immerse the gels with embedded cells in the medium. The medium was changed after 24 h and subsequently every 48 h until the live-dead staining and cell-viability experiments were finished (14 days). As a control hydrogel, 75 µL unmodified gelatin at a concentration of 133 mg/mL and 25 µL of a solution containing cells at a concentration of 4 × 10^6^ cells/mL was mixed and added to the well. As described in a previous study [55], 7.5 µL of transglutaminase was added and carefully mixed with the gelatin solution. As a further control, wells containing cells without gel were prepared.

### 4.6. Statistical Calculations

The statistical analysis was performed using GraphPad Prism v10 software. The statistical difference was determined for an error probability of *p* ≤ 0.05 and marked by asterisks in the figures.

## Figures and Tables

**Table 1:** Functionalization degree of hGel and oxPS.

Sample	Degree of Functionalization
hGel	48.6%
oxHA *	12.5%
oxALG *	20.7%

* Data were converted into the content of aldehyde substitution of the dimeric unit of oxPS. hGel: hydrazide-modified gelatin; oxHA and oxALG: oxidized hyaluronan and alginate.

**Table 2:** Gelation time, storage modulus G’, and loss modulus G” of hydrogels.

Oxidized Polysaccharides	Gelation Time (min)	G’ (Pa) *	G” (Pa) *
oxALG	39.85 ± 3.7	414.78 ± 55.8	9.0 ± 2.2
oxHA	23.08 ± 6.6	521.37 ± 59.9	22.70 ± 6.0

* Gel composition: oxPS:hGel = 1:1. Concentration of oxPS components: 50 mg/mL. Concentration of hGel: 160 mg/mL. Values were determined through the frequency sweep measurements at 1 Hz. Means ± standard deviations, *n* = 3.

## Data Availability

All data and materials are available on request from the corresponding author. The data are not publicly available due to ongoing research using part of the data.

## Supplementary Materials

The following supporting information can be downloaded at: https://www.mdpi.com/article/10.3390/gels10040231/s1, Figure SI-1: 1H-NMR spectrum of native gelatin (upper spectrum) and hydrazide functioanlized gelatin (lower figure). Marked areas highlight signals appearing after the functionalization with hydrazide groups.; Figure SI-2: Degradation study of hydrogels composed of either oxHA or oxALG and hGel of different concentrations. *n* = 3; Figure SI-3: (a) Confocal microscopy pictures of HepG2 cells incubated in EMEM culture medium containing the indi-vidual components of the hydrogel. Photos were taken after 24 hours of incubation. (b) Q-Blue viability assay evaluation of HepG2 cells incubated in the individual hydrogel components with pure EMEM as control. Incubation time 1 and 5 days.
