# Supplementary material for: Functionalized Gelatin/Polysaccharide Hydrogels for Encapsulation of Hepatocytes
Source: Gels. 2024 Mar 28;10(4):231. doi: 10.3390/gels10040231 (PMC11048940; doi:10.3390/gels10040231)
Supplement: Supplementary file 1 [file gels-10-00231-s001.zip › gels-2887724-supplementary.pdf]

gels

MDPI

Supplementary Materials

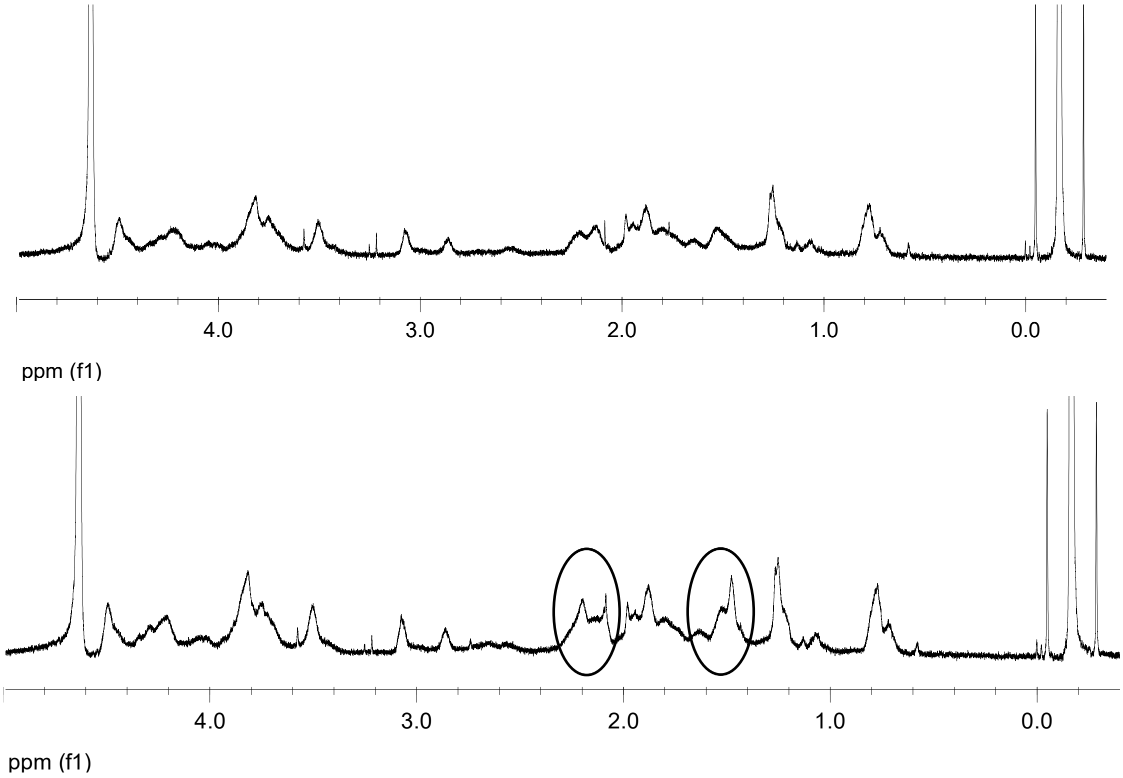

Figure SI-1. ^{1}H-NMR spectrum of native gelatin (upper spectrum) and hydrazide functioanlized gelatin (lower figure). Marked areas highlight signals appearing after the functionalization with hydrazide groups.

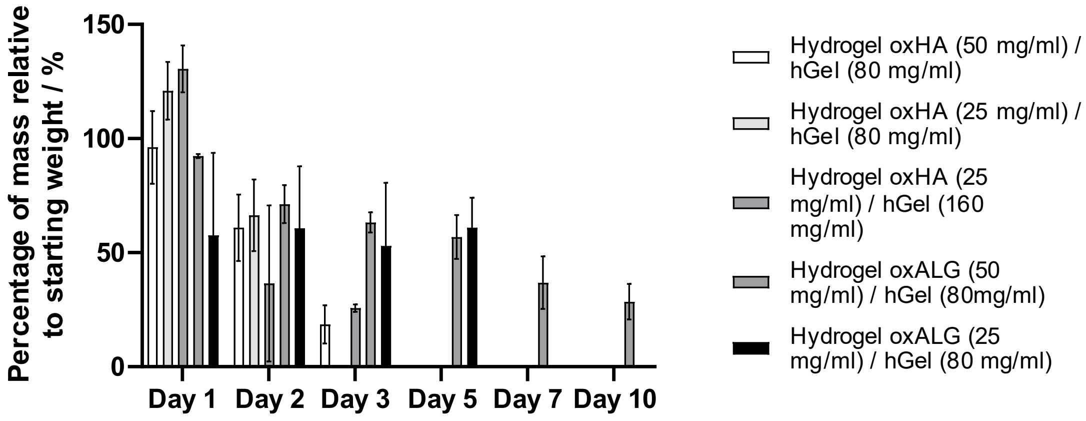

Figure SI-2. Degradation study of hydrogels composed of either oxHA or oxALG and hGel of different concentrations. $n = 3$.

(a)

Medium: EMEM	Medium: EMEM + hGel (160 mg/mL)
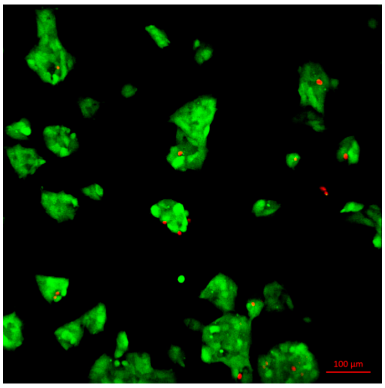	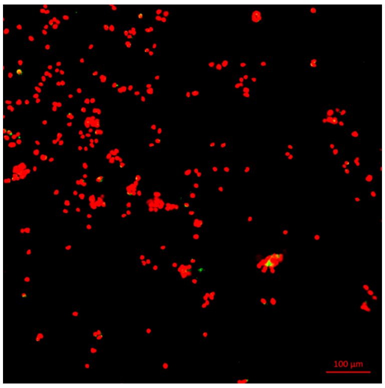
Medium: EMEM + oxALG (50 mg/mL)	Medium: EMEM + oxHA (50 mg/mL)
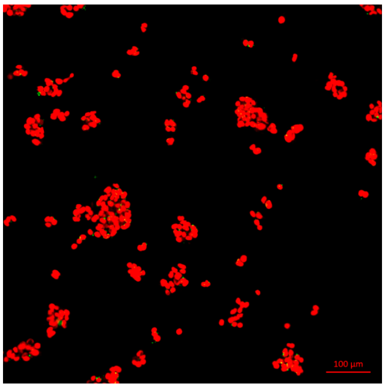	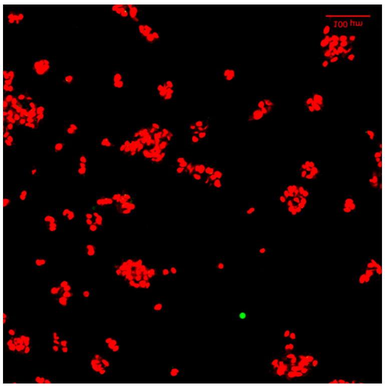

(b)

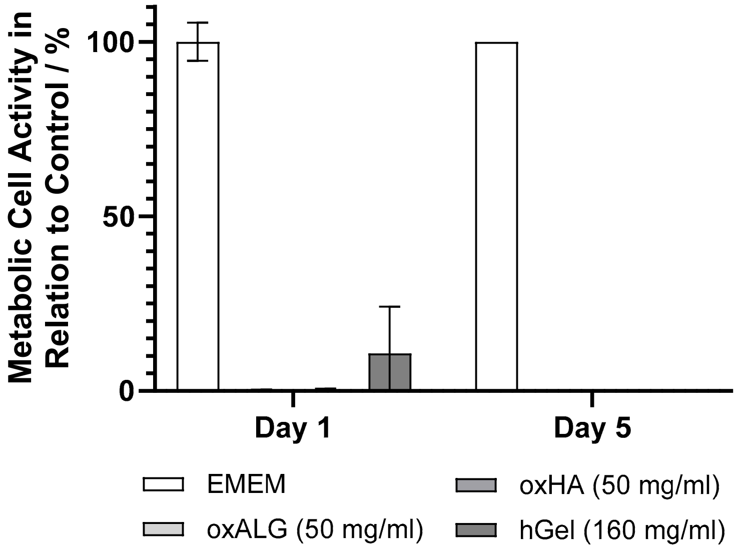

Figure SI-3. (**a**) Confocal microscopy pictures of HepG2 cells incubated in EMEM culture medium containing the individual components of the hydrogel. Photos were taken after 24 hours of incubation. (**b**) Q-Blue viability assay evaluation of HepG2 cells incubated in the individual hydrogel components with pure EMEM as control. Incubation time 1 and 5 days.
